# Recent Techniques in Nutrient Analysis for Food Composition Database

**DOI:** 10.3390/molecules25194567

**Published:** 2020-10-06

**Authors:** Mohd Fairulnizal Md Noh, Rathi Devi-Nair Gunasegavan, Norhayati Mustafa Khalid, Vimala Balasubramaniam, Suraiami Mustar, Aswir Abd Rashed

**Affiliations:** Nutrition, Metabolism and Cardiovascular Research Centre, Institute for Medical Research, National Institutes of Health, No.1, Jalan Setia Murni U13/52, Seksyen U13 Setia Alam, Shah Alam 40170, Malaysia; rathidevinair@moh.gov.my (R.D.-N.G.); norhayati.mk@moh.gov.my (N.M.K.); vimala.rmt@moh.gov.my (V.B.); suraiami@moh.gov.my (S.M.); aswir@moh.gov.my (A.A.R.)

**Keywords:** Food Composition Database (FCD), sample preparation, recent techniques

## Abstract

Food composition database (FCD) provides the nutritional composition of foods. Reliable and up-to date FCD is important in many aspects of nutrition, dietetics, health, food science, biodiversity, plant breeding, food industry, trade and food regulation. FCD has been used extensively in nutrition labelling, nutritional analysis, research, regulation, national food and nutrition policy. The choice of method for the analysis of samples for FCD often depends on detection capability, along with ease of use, speed of analysis and low cost. Sample preparation is the most critical stage in analytical method development. Samples can be prepared using numerous techniques; however it should be applicable for a wide range of analytes and sample matrices. There are quite a number of significant improvements on sample preparation techniques in various food matrices for specific analytes highlighted in the literatures. Improvements on the technology used for the analysis of samples by specific instrumentation could provide an alternative to the analyst to choose for their laboratory requirement. This review provides the reader with an overview of recent techniques that can be used for sample preparation and instrumentation for food analysis which can provide wide options to the analysts in providing data to their FCD.

## 1. Introduction

Food composition database (FCD) or also referred to as food composition tables (FCT) are the basis for almost everything in nutrition. FCD or FCT, are data that provide the nutritional composition of foods. The data are normally derived from quantitative chemical analysis of representative samples of foods and beverages [1]. Data that are provided in the FCD are macronutrients which are required in larger quantities in the body like carbohydrates, lipid, proteins and also micronutrients, which are required in smaller quantities which include vitamins and minerals [2]. The number of nutrients included in the FCD depending on the requirement by regulators or countries involved in the compilation of FCD.

Relevant, authentic and up-to-date food composition data are the basis and of fundamental importance in many aspects of nutrition, dietetics and health, but also for other disciplines such as food science, biodiversity, plant breeding, food industry, trade and food regulation [3]. FCD has been used extensively in many areas, including for nutritional analysis or assessment of nutrient intakes, prescription of therapeutic diets, nutrition labelling, research into diet-disease relationships, national food and nutrition policy, nutritional regulation of the food supply and planning of nutrition intervention program [4].

One of the criteria for a comprehensive FCD is that the data produced by the compiler should have analytical quality. Ideally, data should come from original analytical data from thoroughly inspected sources. High quality analytical data should come from methods that have been shown to be reliable and appropriate to the food matrix and nutrient to be analyzed. The methods used must apply proficiency testing and evidence of this proficiency testing must be shown to assure data quality. The analyst and the laboratory involved in the analysis should meet criteria of good laboratory practice (GLP). The outgrowth of a reliable new or improved method for measuring nutrients may create the need for analysis (or reanalysis) of existing foods that are important in the food supply or that are known or suspected to be good sources of the nutrient concerned [4]. The representative food samples that are collected during sampling should be handled properly in order to preserve the sample integrity during storage and sample preparation [3]. The sample preparation step is one of the most critical steps in the analytical process. Sample preparation has a fundamental impact on laboratory throughput and analytical performance. Any errors within the sample preparation process will undermine the quality of data at all subsequent stages of the analysis [5].

Reliable data on the nutrient composition of foods is crucial and can only be obtained firstly, by a careful performance of appropriate, accurate and precise analytical methods. Secondly, the choice of the appropriate methods carried out by a trained analyst should follow by a quality assurance schemes. This is a crucial element in ensuring the quality of the values in a food composition database. Every analyst should consider three important criteria’s in choosing the right methods for FCD. Firstly, predilection to the methods that have been recommended or adopted by international organizations such as Association of Official Analytical Chemists (AOAC). Secondly, predilection to the methods for which reliability has been established by collaborative studies involving several laboratories either locally or internationally. Lastly, predilection to the methods which applicable to a wide range of food types and matrices rather than those focused only for specific foods [6].

The analytical method selected also needs to have adequate performance characteristics. Buttner and co-workers summarized these as reliability criteria (specificity, accuracy, precision and sensitivity), ruggedness and practicability criteria (speed, costs, technical skill requirements, dependability and laboratory safety) [7]. The performance characteristics need to be performed by every analyst prior to use the techniques for generating data for FCD.

**Figure 1:**
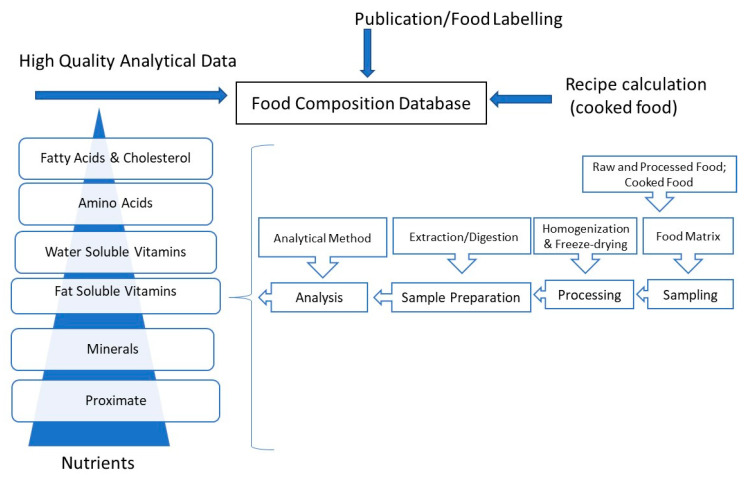
Overview of process in analyzing food for Food Composition Database.

There are continuous developments in analytical chemistry which enable FCD’s experts to use newer techniques which can provide more robust, faster, solvent-less, higher extraction rates, inexpensive procedures and automated system for the updates of FCD in their own settings. This review provides the reader with an overview of recent techniques available that can be used for detection of important nutrients in food samples which can provide alternatives to their existing traditional methods (Figure 1).

## 2. Proximate

The values of the individual macronutrients in food samples can be determined with the proximate analysis. The proximate composition of foods includes moisture, ash, lipid, protein and carbohydrate contents [8]. These values are being declared as nutritional facts which are usually being shown on the labels of the end food products, but they are also being determined during the food production process. Analyses used may be rapid methods for quality control (QC) or more accurate but time-consuming official methods (Table 1).

### 2.1. Moisture

Water is one of the most crucial components of many food products [23]. According to authors [24]; quality, shelf life and sensory features of the product depend on the quantity of water stored in it. Therefore, the water content should be precisely determined and controlled during the product manufacturing process.

Infrared radiation (IR) is used in many moisture analyzers, such as halogen moisture analyzers which are used to produce infrared radiation from a halogen lamp. IR radiation wavelength emitted by the infrared radiator is strictly conditioned by the IR radiator temperature [9]. The weight of the sample is measured and recorded continuously and once it becomes constant the drying is stopped. The difference in the weight of the sample at the end of drying is used to calculate the moisture percentage. Halogen lamps are used in preference to ordinary infrared generators as they are much lighter and therefore achieve maximal heat output very fast and allow excellent control of the heating process as they heat up and cool down rapidly. They also distribute the heat uniformly over the sample surface which promotes good reproducibility. The infrared radiation in such devices is absorbed by the moisture analyzer and this further reduces the time taken to heat up the sample. The infrared and halogen moisture analyzers are destructive to the sample. However, because of the speed of analysis, this technique is suited for qualitative in-process use.

Microwave radiation method is also an extremely rapid method of drying up a sample, but the temperatures achieved are very high, making it suitable only for very thermostable materials. Larger samples can be used but the level of control of heating is reduced. Like the infrared method, the sample is typically destroyed by the analysis. It is also not useful if the moisture content is below 2%. An example of the use of the microwave method is a moisture content meter developed by the United States Department of Agriculture, which was integrated into a convection drying system for peanuts. This allows real-time moisture determination of a peanut kernel without shelling the peanuts [25].

Near infrared reflectance (NIR) method, for example, have been widely applied for the analysis of cereal grains [11]. The method requires calibrating with a large number of samples with moisture values measured by conventional methods to develop the analytical equations. Nuclear magnetic resonance (NMR) [12] method also require detailed calibration and are of the greatest value in measuring the distribution of water in foods, identifying the forms of water in meats and herbal products.

### 2.2. Protein

For many years, the protein content of foods has been determined on the basis of total nitrogen content, while the Kjeldahl method has been almost universally applied to determine nitrogen content. Recently, an automated instrumental technique has been developed which is capable of rapidly measuring the protein concentration of food samples. This technique is based on a method first described by a scientist called Dumas over a century and a half ago. It is beginning to compete with the Kjeldahl method as the standard method of analysis for proteins for some foodstuffs due to its rapidness [13].

### 2.3. Total Fat

The commonly used techniques are Soxhlet analysis and acid/alkaline hydrolysis [26]. A new method based on an innovative microwave-assisted extraction (MAE) technique allows the determination of total fat in cheese samples. MAE method is statistically equivalent to the other method, showing good performance indicators (limit of quantification; LOQ = 0.248%, limit of detection; LOD = 0.087%, expanded uncertainty; U = 2.65%) and allows the determination of total fat in 12 cheese samples simultaneously in 100 min [14].

### 2.4. Total Dietary Fibre (TDF)

Rapid Integrated Total Dietary Fibre (RITDF) (AOAC 2017.16) is the current technique for TDF; and closely resembles AOAC 2009.01 [20]. This method addresses the minor limitations that have been identified in the McCleary Method (AOAC 2009.01) and is the only method that accurately measures all components of TDF (including all forms of resistant starch) [20]. Several problems/challenges that became evident with the initial procedure have been resolved; enzyme levels have been optimized allowing an incubation time (4 h) consistent with human ileostomy transit time; problems associated with measurement of fructo-oligosaccharides (FOS) have been resolved by using a different high performance liquid chromatography (HPLC) system, meaningful analytical results have been obtained for phosphate-crosslinked starch.

### 2.5. Ash

Ash is inorganic residue remaining after water and organic matter have been removed by presence of oxidizing agents, which provides a measure of total minerals within a food. The most commonly used process is dry ashing. A muffle furnace is used to burn down the sample. The temperature of the chamber is maintained to approximately 600 °C. During this process, most of the minerals get changed into phosphates, sulphates and oxides. Due to the presence of some volatile materials in the sample, the test results are prone to being inaccurate. Therefore, other testing methods are preferred when materials like lead, mercury and iron are present in the sample. The Attenuated total reflection Fourier-transform infrared spectroscopy (ATR-FTIR) method requires a small drop/amount of sample on the ATR base-plate, being much faster than traditional techniques, allowing potential applications for simultaneous determination of sulphur, nitrogen, and ash contents for routine analysis of selected plant tannins by FTIR data [21].

### 2.6. Total Sugar

Sugars refer to all mono- and disaccharides present in food. Glucose, galactose, and fructose are common monosaccharides, whereas common disaccharides include lactose, maltose, and sucrose. Quantification of sugars are currently determined via several methods. This include enzymatic method that measures sucrose hydrolysis and phosphorylation of glucose and fructose [27] or measuring absorbance increase according to standard assay of sugar kit [28]. In addition to this, HPLC technique coupled with refractive index detector (RID) [29] or evaporative light scattering detector (ELSD) are also used [30]. HPLC-ELSD presents numerous advantages in terms of its sensitivity, stability and compatibility with gradient elution, compared to HPLC-RID [27,30]. Recent advances in gas chromatography-mass spectrometry (GC-MS) equipment and columns have resulted in this method becoming useful in compositional and structural analysis of monosaccharides, oligomers and polymers, especially in the environmental and life sciences fields [22].

### 2.7. Carbohydrate

The carbohydrate content of a food can be determined by calculating the percent remaining after all the other components have been measured, as per the formula below. In these instances, an energy factor of 17 kJ/g (4 kcal/g) should be used [31].

%carbohydrates = 100 − %moisture − %protein − %lipid − %ash(1)

## 3. Minerals

Minerals and trace elements are naturally occurring inorganic substance that account for about 4% of total human body mass. Its serve as materials and regulators in numerous biological activities in body structure building and needed for good health. Approximately 30 elements have been recognized as essential. Minerals are grouped into two main categories: major minerals and trace minerals. Major minerals (calcium, potassium, magnesium, phosphorus, sodium, sulphur) are required in higher quantities in daily diet, while trace minerals (chromium, iron, copper, iodine manganese, molybdenum, selenium, zinc) are only needed in smaller amounts [32].

Mineral analysis in food samples generally requires sample preparation and can be carried out manually or by using automated or mechanised processes [5]. Food samples need to be converted into liquid solution or digested form to enable the samples to be used for analysis with the desired techniques. These includes dissolution or homogenisation step (blending, mixing, grinding or slurry preparation) of the sample, then followed by a collection of a representative test portion. There are two ways the samples will be digested which remove a large number of potential interferences through acid or alkaline-assisted hot digestion with or without high pressure. The final sample digest is commonly diluted in an acidic or alkaline aqueous solution and, as a result, easily amenable to any analytical technique [32,33].

Nowadays, the latest techniques for digestion usually use open vessel which atmospheric pressure digestion is a common approach to sample preparation [33]. Another popular approach of digestion is by using microwave assisted-digestion (MW-AD) and microwave induced-combustion (MIC) [34] (Table 2). Once the organic matrix has been removed, the inorganic constituents can be measured using a variety of instrumental techniques. Instrumental techniques normally offer an increase in the speed of analysis, automation, good precision and accuracy. For minerals, there are several alternative analytical methods that are available which can give comparable results. Basically, the instruments being used for the analysis of minerals are still the same as ten years ago. Most improvements are on the technologies used in the extraction or sample preparation and instrumentations which normally can help to reduce the interference, enhance sensitivity of the technique and speed of the measurement.

### 3.1. Atomic Absorption Spectrometer (AAS)

AAS is an analytical technique to quantitate the concentration from part per-million (mg/L) level to part-per-billion (µg/L) level of multi-elements in all types of samples including food samples using the absorption of light by free atoms in the gaseous state. The analyte concentration is determined from the amount of absorption of specific light at a suitable wavelength [61].

AAS instrumentation includes either flame or graphite furnace atomizers. Flame atomizers commonly use air–acetylene for atomization of many analytes at a temperature of 2300 °C, whereas nitrous oxide acetylene is used for selected analytes that require hotter flames of 2900 °C. In contrast, graphite furnace atomizers use a flameless technique where the graphite tubes are heated electrically. The graphite furnace technique is mostly automated compared to flame AAS methods and generally about 100–1000 times more sensitive than flame AAS under any given radiation sources [61].

The latest AAS uses fibre optic technology that produces a fully enclosed optical system. The optical system improves light throughput for better detection limits. The new light path also helps to reduce the size of the instrument. It also uses a stacked design where both flame and graphite furnace can be used on the same instrument. This involves a titanium burner that can be easily removed for different analyses. It features a double beam design for quick start-up and long-term stability without the need for recalibration [62].

The AAS instrument can configure either deuterium or longitudinal Zeeman background correction to suit any particular analysis. Deuterium background ensures the best sensitivity and accuracy over a wide wavelength range [63]. The longitudinal Zeeman design allows the use of transverse heated graphite tube (THGA), which can significantly reduce matrix effects [64]. AAS is also equipped with a colour furnace camera that allows better sample monitoring. In addition, no gas line connections are required due to a new mixing chamber design. It can also utilize an air purge to clean the inside of the instrument to eliminate corrosive vapours. The instrument can also save running costs by determining the concentration of all elements from a single aspiration sample. The matrix modification technique using chemical modifiers is a very important feature in the concept of trace metal determinations with very minimal interference. The chemical forms and therefore the physical properties of the element studied or the matrix can be changed by the addition of a suitable reagent (matrix modifier) in excess to the sample and standard reference solutions [36,65,66,67,68].

Slurry sampling is a technique of direct sample preparation and known to be capable of minimizing the reported drawbacks of manual and automated sample digestion. This analytical technique is suitable only for graphite furnace AAS [38,69]. There was new application in analysing food samples using AAS such as determination of meat and baby food samples [37], infant formula [40], vegetables [39], fruit juices [35], fish fillet [41] and vegetable oils [42]. The development and progress of several AAS technique and application in various food matrices is summarized in Table 2.

### 3.2. Microwave and Inductively Coupled Plasma-Optical Emission Spectrometry/Atomic Emission Spectrometry (ICP-OES/AES))

OES/AES is based on the principle that when energy is applied to a samples/molecule in the form of light or heat, it will be dissociated into an atom but also cause collisional excitation (and ionization) from a lower energy state to a higher energy state. However, at a higher energy state, the molecules are unstable and decay to the lower energy state and thus emitting radiations in the form of photons. The specific wavelengths of emitted photons are recorded in the emission spectrometer and used to determine the concentrations of the elements of interest [70].

ICP is the most common excitation process which requires a plasma torch of concentric quartz tubes to induce excitation of samples. The process requires argon gas and radiofrequency generator which produces the plasma and the sample particles entering the plasma then undergo desolvation, dissociation, atomization and excitation [70]. In the microwave-induce plasma method, a microwave generator will produce a microwave that travels through a cable and is focused via a tuning system where a torch sits in the centre of the cavity. Nitrogen gas is used to spark the plasma. Cheese was analysed using this method [71].

AES has been widely used for the analysis of accurate and precise in food composition which enable consumers to make their food choices based on dietary reference intakes [72]. ICP-OES/AES was mostly used to determine the geographical origin of honey [48], wines [49], cumin [45], vinegar [50], coffee beans [46], baby foods, vegetables, milk powder [47] for minerals such as Mn, Zn, P, Fe, Cu, Rb, Mo, Ba, Sr and Ni. Slurry sampling had also been applied for the determination of minerals in sugarcane with ICP-OES [43]. Soft drinks were successfully analysed for several elements [44] (Table 2).

### 3.3. Inductively Coupled Plasma-Mass Spectrometer (ICP-MS)

ICP-MS is a powerful multi-analyte analytical technique used for the quantitation of metals and non-metals in a wide range of digested sample at low concentration, typically parts per billion (ppb) or per trillion (ppt) level. Samples will be converted to neutral components in high temperature plasma and identified based on mass to charged ratios. There are four mains steps involved in ICP-MS analysis such as sample introduction, aerosol generation, ionization by an argon plasma source, mass discrimination and the detection system. ICP-MS is a sophisticated and highly reliable technique due to its excellent sensitivity, wide analytical working range (up to 9 orders of magnitude), multi-element characteristics, short analysis time, reduce sample-handling requirements, minimised potential analytical errors and isotopic capabilities [32,55].

There are many complex samples matrix have been successfully analysed using ICP-MS such as honey [56], rice [57], cow, goat, buffalo, yak and camel milk [55], meat [51], spices and aromatic herbs [58], honeydew honey [52], dairy milk and plant based milk [54] and maize [59]. Hyphenation techniques such as size-exclusion chromatography (SEC-ICP-MS) for the separation of samples and analyse using ICP-MS has been applied for analysis of metals such as Fe, Zn and P in vegetables [53] (Table 2).

### 3.4. Energy-Dispersive X-Ray Fluorescence (ED-XRF)

A non-destructive energy-dispersive X-ray fluorescence (ED-XRF) spectrometry is a nuclear analytical technique was used for macro elements calcium and potassium analysis in food samples such as fruits, vegetables [60] and cumin spice [45]. Good comparability with Neutron Activation Analysis (NAA) and AAS showed by statistical results confirming no significant difference between EDXRF and both NAA/AAS methods [60].

## 4. Fat Soluble Vitamins and Carotenoids

Fat-soluble vitamins (FSVs) and carotenoids are soluble in non-polar solvents. FSVs include vitamin A (retinol, retinyl acetate, retinyl palmitate), vitamin D (D_2_: ergocalciferol, D_3_: cholecalciferol), vitamin E (α-, β-, γ-, δ-tocopherols and tocotrienols) and vitamin K (K_1_: phylloquinone, K_2_: menaquinone). Carotenoids are a group of organic compounds that are composed of 2 or more units of hydrocarbons; and are categorized into two sub-groups known as carotenes and xanthophylls. Carotenes (α-carotene, β-carotene and lycopene) are made up entirely of carbon and hydrogen, whereas in presence of oxygen they are called xanthophylls (β-cryptoxanthin, lutein, astaxanthin and zeaxanthin) [73,74]. In general, vitamins are highly unstable and degrade rapidly under several conditions such as heat, oxygen, light, moisture and certain pH [75]. The sufficient level of vitamin consumption should be met as per recommended level, and as such accurate and sensitive analytical techniques are necessary.

### Sample Preparation and Analytical Technique

Vitamin analysis, generally encounters many difficulties accounted by various factors that include chemical instability, intra- and inter-group chemical heterogeneity, low concentration, matrix effects as well as interaction with other food components [75]. The AOAC method for analysis of specific vitamins is reported in early years and has been continually improvised. Determination of vitamin A (retinol) in milk-based infant formula (AOAC 992.06) [76], determination of vitamin D_3_ (cholecalciferol) in ready-to-feed milk-based infant formula (AOAC 992.26) [77], vitamin E activity in milk-based infant formula (AOAC 992.03) [78] and analysis of trans-vitamin K_1_ (phylloquinone) in ready-to-feed milk-based infant formulas (AOAC 992.27) [79], via liquid chromatography are among the available official methods.

In general, fat soluble compounds are extracted via alkaline saponification followed by liquid-liquid extraction (LLE) using organic solvents. Saponification is performed under alkaline condition at ambient/elevated temperature with antioxidants and under an inert atmosphere. Hot saponification can be applied for individual or simultaneous extraction of carotenoids along with vitamin A, D and E; however, it is deemed not suitable for vitamin K. Vitamin K homologues rapidly degrades in alkaline conditions under high temperature, hence overnight cold saponification is applied where digestion is performed longer, in dark environment [80]. Conventional techniques are prone to degradation, low recoveries, consumes large amount of solvent, as well as laborious and time-intensive [75]. Thus, many researches have been focused on the development of compressed fluid-based as well as miniaturized extraction techniques that are environmental-friendly [75].

One such alternative technique is known as pressurized liquid extraction (PLE) or accelerated solvent extraction (ASE) that utilizes solvents at elevated temperature and pressure to increase isolation yields, reduce extracted volume and extraction time. An added benefit of this technique is the usage of stainless-steel extraction cell that generates oxygen and light-free environment that minimizes the degradation rate. This technique was reported for vitamin K_1_ extraction from fruits and vegetables where C_18_ was used as a sorbent using heptane and ethyl acetate [81]. However, PLE also encounters partial shortcomings in relation to high cost and variability of extracted volumes [75]. Apart from PLE, dispersive liquid-liquid microextraction (DLLME) is the miniaturized evolution of LLE. DLLME can either be applied alone or in combination with preliminary digestion for FSV extraction from a large variety of foods. [75].

Along with various developments of extraction techniques, selection of the suitable chromatographic technique is also equally important. Liquid chromatography (LC) in all variants, including HPLC, ultra-high-performance liquid chromatography (UHPLC), nano-liquid chromatography (nano-LC), two-dimensional liquid chromatography (2D-LC) is the most suitable analytical method for determination of FSV and carotenoids in various matrices. These techniques are also related to its versatility in terms of the chromatographic modes such as normal phase (NP), reversed-phase (RP), non-aqueous reversed-phase (NARP), and even the column packings. In addition, LC techniques are also equipped with various detectors [ultraviolet/visible (UV/Vis), diode array (DAD), photo-diode array (PDA), fluorescence (FLD), electrochemical (ED), mass spectrometry (MS)] that offer vast technical solutions for vitamin characterization in complex food matrices. Among the various detectors, MS is deemed to be highly selective and portrays high ability to identify low levels of vitamins and also to perform profiling studies [75].

Over the years, a number of studies have been published reporting on the possible approach of extraction and detection of single as well as multiple vitamins within one analytical run. The various techniques that have continuously evolved over the years along with its advantages are summarized in Table 3. Selected techniques, with focus on simultaneous analysis are described in detail below. A most recent study reported in the year 2020 highlighted the application of dispersive solid-phase extraction (DSPE) using newly synthesized polymeric material comprised of tetracycline-grafted polyacrylamide polymer (PAA-T) and HPLC-DAD for simultaneous analysis of vitamin A and E in milk and egg yolk samples. The technique was deemed to be simple, sensitive and cost-effective. The application of tetracycline as solid phase sorbent is useful as it provides good zones for the interaction of vitamin molecules with solid material [82].

Another literature also reports on simultaneous detection of vitamin A and E esters along with β-carotene in infant formula and fortified milk powders using NP-HPLC with selective dual-channel FLD and DAD. The highlight of this research is the elimination of saponification procedure where it applies protease enzyme to remove vitamin encapsulation and facilitate vitamin partition into hydrocarbon solvent. Extraction via protease digestion is faster with adequate precision and recovery, compared to conventional technique. Furthermore, other naturally present esters (retinyl propionate, α-tocopheryl propionate) could be utilised as an internal standard when the sample preparation excludes saponification step [91]. Apart from vitamin A, E and carotenoids, concurrent analysis with vitamin D and K have also been reported in simple matrices. However, as these vitamins are usually present at trace levels, establishing an accurate method in complex matrices can be challenging. As an effort to address this, a study in 2019 showcased simultaneous detection of vitamin D and K in cereal and flour products via simplified saponification in combination with solvent extraction followed by UHPLC-DAD equipped with on-line solid phase extraction (SPE) system that proves advantageous for further sample clean-up and improved detection [93].

Among majority studies that are focused on LC techniques, another technique known as Ultra performance convergence chromatography (UPC^2^) was also reported to successfully detect FSV and carotenoids. UPC^2^ separation uses compressed carbon dioxide as the primary mobile phase with sub-2-μm particle columns in an advanced chromatography system that provides rapid separation with high reproducibility, efficiency, and selectivity. Application of this technique was attempted in a study to establish detection of seven FSV compounds (retinyl acetate, retinol, α-tocopherol, D_2_, D_3_, K_1_, K_2_) and three carotenoids (lutein, lycopene, β-carotene) in cooking oil samples where it was achieved on separate analytical runs within 8 and 3 min, respectively. Sample preparation include simple dilution in methyl tertiary-butyl ether (MTBE) followed by sonication and filtration prior to analysis. UPC^2^ technique is environmental-friendly, cost-effective, with improved repeatability and shorter time compared to HPLC [74].

Supercritical fluid chromatography (SFC) also applies the same concept as UPC^2^. A recent study in the year 2018 reported on detection of nine analytes (retinyl acetate, retinyl palmitate, retinol, α-tocopherol, α-tocopheryl acetate, cholecalciferol, ergocalciferol, phylloquinone, menaquinone-4) in infant formulas, infant cereals, adult nutritionals and frozen mixed meals. Sample pre-treatment involves direct solvent extraction using enzyme-assisted matrix disintegration and methanolic protein precipitation, followed by separation and detection via SFC with detection by tandem MS (MS/MS) in positive atmospheric pressure chemical ionisation (APCI) mode (SFC-APCI (+)-MS/MS). Vitamin A, E and K are determined via direct injection of the extract, while vitamin D undergoes derivatization prior to detection. The method shows enhanced safety and reduced cost compared with previous methodologies, with the potential to be applied for more complex matrixes [92].

In addition to chromatography techniques, another recent sensation is the application of sensor-based technologies for detection of FSV. A study by Sys and colleagues in 2017 reported on the application of ED by square wave anodic stripping voltammetry (SWASV) for detection of vitamin E in margarines and edible oils. The method is based on extraction into silicone oil; that acts as a lipophilic binder of glassy carbon paste electrode (GCPE) followed by ED. In comparison with the reference method of RP-HPLC-UV, electrochemical approach is believed to provide an alternative option for easy sample preparation, as well as rapid and cheaper analysis [88]. Similarly, in another study by the same research team, determination of vitamin K_1_ was attempted using ED by square wave adsorptive stripping voltammetry (SWAdSV) in extra virgin olive oil. The method here is based on adsorptive accumulation onto solid glassy carbon electrode (GCE) surface followed by ED. Among the various benefits of this technique include minimal solvent consumption, cost as well as easier sample preparation [90].

## 5. Water-Soluble Vitamins

Water Soluble Vitamins (WSVs) are essential micronutrients and includes vitamin C and B complex vitamins such as thiamine (vitamin B_1_), riboflavin (vitamin B_2_), niacin (vitamin B_3_), pantothenic acid (vitamin B_5_), pyridoxine (vitamin B_6_), biotin(vitamin B_7_), folic acid (vitamin B_9_) and cobalamins (vitamin B_12_) [94]. WSVs act mainly in the metabolism of carbohydrates, lipids and proteins, and involved in physiological roles such as maintenance of healthy muscle, skin, eyes, hair and liver [95]. As the human body is unable to synthesize and store WSVs (except vitamin B_12_), they must be obtained through daily intake from food [96]. Analytical method for analysis of WSVs in food samples is complex due to low and different concentration, interaction with other compounds such as protein, complexity of food matrices and poor stability of vitamin solutions [95,97]. Recently, interest in research on the development of a fast, reliable and economical analytical method for WSVs has increased. A number of current improved techniques were developed. The methods used and the advantages of current improved techniques for WSVs are summarized in Table 4.

### Sample Preparation and Analytical Technique

There are several recent sample preparation techniques for vitamin C analysis, including acid extraction and PLE. Both L-ascorbic acid (L-AA) and dehydroascorbic acid (DHAA) show the biological activity of vitamin C [102]. The acid techniques have been used widely for L-AA extraction, involve agitation of the sample in acid solution, followed by centrifugation [98,99,100,101,102,103,119,120]. Acid solutions help to stabilize vitamin C. The most frequently used acids are metaphosphoric acid (MPA) and ethylenediaminetetraacetic acid (EDTA) [98,99,100,101,102,120]. PLE technique can be considered an alternative extraction technique for L-AA, since it uses only water as solvent and high extraction rate of vitamin C can be obtained due to the combination of pressure and temperature in absence of oxygen and light [100].

The simplest analytical method for quantification of vitamin C is the official AOAC titrimetric method [121]. However, disadvantages of titration method were the exposure to light and oxygen during the analysis that can lead to L-AA degradation and the interference at the turning point of the indicator that can cause an overestimation of the LAA content [122]. At present, HPLC with DAD or PDA detectors is the most widely used analytical method for vitamin C. HPLC with PDA detection is a sensitive method for the simultaneous analysis of ascorbic acid (AA) and total vitamin C [98]. UHPLC with PDA detector has also been used for the determination of vitamin C [99,100]. UHPLC method is faster, more sensitive, use less solvent and more environmentally friendly than the conventional HPLC method [99,100].

UHPLC coupled to MS technique has been also reported for determination of L-AA and DHAA [101]. The UHPLC-MS technique enables the direct detection of both analytes and has significant advantages of higher sensitivity and selectivity [98]. Voltammetric sensor technique has also been demonstrated to be a fast, simple, selective and sensitive method for the determination of AA in fruit juices [119]. Capillary electrophoresis (CE) is another technique used for the determination of vitamin C. The advantages of CE method using micellar electrokinetic chromatography (MEKC) mode were it implies minimal sample preparation and reagent consumption and being environmentally friendly [120]. UV-visible spectrophotometer method has also been developed for determination of vitamin C. This method has been preferred because it is simple and fast compared to titration method [103]. The polarographic method is another technique that allowed easier and faster determination of vitamin C [102].

The commonly used extraction method for B complex vitamins analysis, including protein precipitation [108,113,117,123], acid hydrolysis and/or enzymatic treatment [109,114,118] and SPE [112,116]. Protein precipitation is a common method used for extraction of B complex vitamins from samples rich in protein like milk. In a study by Schmidt et al. [123], different protein precipitation reagent have been used include hydrochloric acid (HCI), trichloroacetic acid (TCA) and perchloric acid (PCA). HCI was found to be the most suitable for the study of native B_6_ and B_2_ vitamers in cow’s milk. Acid hydrolysis and/or enzyme treatment have been used to release bonded vitamins into their free form and for the reduction of number of possible vitamers [97]. Caprioli et al. [118] made a comparison of four different extraction samples procedures: acidic hydrolysis, acidic hydrolysis plus peptide precipitation, acidic plus enzymatic hydrolysis and enzymatic hydrolysis and it showed that acidic hydrolysis (0.1 N HCI), gave excellent results, with high recoveries and shorter time extraction for the analysis of vitamins B_2_ and B_3_ in anchovies. On the other hand, DSPE showed more advantages in clean-up technique compared to general SPE since it does not require conditioning of sorbent and consumes less solvent [116].

The current international methods for B-vitamins are based on microbiological assays. However, the disadvantage is time-consuming [124]. Recently, most widely used analytical method for B complex vitamin analysis are HPLC coupled with UV [110,116], FLD [104,106,108,123], DAD [115] and ED [109] detectors. However, the application of LC-MS/MS method for vitamins analysis has been increased due to its higher sensitivity and specificity especially in complex matrices allowing multivitamin analysis [117,118]. Besides that, LC-MS/MS is also possible to identify very low concentration of different vitamins in food samples [97] and saving time and cost [94]. CE with MEKC mode technique has been used for simultaneous determination of B complex vitamin in energy drinks, sport drinks and fruit nectars [120]. The advantages of this method are minimal sample preparation and low reagent cost. Microchip electrophoresis (MCE) coupled with laser-induced fluorescence (LIF) is one of recent technique for B complex vitamins analysis in food. MCE offers significant advantages such as miniaturization, reduced reagent consumption and fast operation [107].

## 6. Amino Acids

Amino acids are the building block of protein. Protein is composed of twenty amino acids, known as essential (EAAs) and non-essential amino acids (NEAAs). There are nine EAAs or indispensable amino acids (IAAs) which include histidine (His), isoleucine (Ile), leucine (Leu), valine (Val), lysine (Lys), threonine (Thr), phenylalanine (Phe), methionine (Met), and tryptophan (Trp) [125]. Three of the nine EAAs (Leu, Val and Ile) are the branched-chain amino acids (BCAAs) [126]. These EAAs cannot be synthesized by vertebrates including human and are provided through diet. Only plants can synthesize these amino acids [127]. The other amino acids; alanine (Ala), arginine (Arg), asparagine (Asn), aspartate (Asp), cysteine (Cys), glutamic acid (Glu), glutamine (Gln), glycine (Gly), proline (Pro), serine (Ser) and tyrosine (Tyr) belong to nutritionally NEAAs [128]. Contrasting with the EAAs, the NEAAs can be synthesized by humans and plants [127]. These amino acids are involved in various biological functions including as gene expression regulator, neurotransmitters, for oxidative defense and as an anti-hypotensive factor [129].

In foods, amino acids are present in the free-form, in peptides or proteins. Free-form amino acids are mostly found in beverages (fruit juices and wine), vegetables and fruits. γ-aminobutyric acid (GABA), ornithine (Orn), citrulline (Cit) and β-alanine (β-Ala) are among the free-form amino acids commonly found in foods. The free-form amino acids can be adequately extracted from food matrices using a homogenizer, centrifuged and analyzed directly after [130,131]. Amino acids bound in peptides and proteins however, need to be hydrolyzed using acid or alkaline to breakdown the peptide bond prior to analysis [132]. The determination of amino acids in foods is important to identify the various types of amino acids present so that better choices can be made by the consumers. The analysis of amino acids comprises of several steps; sample preparation (hydrolysis of protein to liberate amino acids), separation of individual amino acids, detection and quantification of the amino acids [133]. The AOAC has proposed various methods using different kinds of instruments to analyze amino acids in food samples depending on the types of amino acids and the food matrices. In the early years, the common methods of analyses were using a spectrophotometer for fruit juices/lemon juice (AOAC 965.31) and ion-electron chromatography (IEC) for animal feed and foods (AOAC 985.28) [134]. In recent years, new methods have been developed using other instruments such as the liquid chromatography for foods/adult/paediatric nutritional formula, baby foods/infant formula (AOAC 2018.06 and 2019.09) [135,136]. Advancement in the analysis of amino acids is moving towards the usage of chromatography techniques such as HPLC, UHPLC, GC and CE. The various methods used recently to analyse amino acids in foods are summarised in Table 5.

### Sample Preparation and Analytical Technique

Amino acids in foods are from a diverse group of compounds and therefore, different hydrolysis procedures are needed to extract all the amino acids due to their variability in interacting with certain chemicals. Previously, the AOAC proposed standardized official methods for protein hydrolysis using three chemicals (HCL hydrolysis, performic acid peroxidation followed by HCL hydrolysis and NaOH hydrolysis) [152]. The use of HCL extracted several amino acids namely Asp, Ser, Glu, Gly, His, Arg, Thr, Ala, Pro, Tyr, Val, Iso, Lys, Ile, Leu and Phe [153]. Sometimes acid hydrolysis was performed with a presence of reducing agents such as phenol, thiols and tryptamine to prevent halogenation and degradation of specific amino acids [154]. Other amino acids, Cys and Met are oxidized to cysteic acid and methionine sulfone (stable compounds) using performic acid to prevent degradation during acid hydrolysis. Alkaline hydrolysis (NaOH) is replaced by acid hydrolysis (HCL) for the determination of Trp in the samples to prevent Trp destruction [155]. In some cases, a microwave is used in conjunction with the acid and alkaline hydrolysis methods to improve efficiency [156,157]. Possibly the best method of hydrolysis involves the use of enzymes because it does not affect most of the amino acids. However, due to the difficulties involving when using enzymes, their applications have been very limited [158]. Recent method developments for amino acids hydrolysis from food protein have shown promising results of using aqueous organic solvents such as ethanol [149] and methanol [159] to replace the use of corrosive acid and alkaline.

Free amino acids from the samples or hydrolysis process are either derivatized or underivatized before being detected and quantified using various analytical techniques. The purpose of derivatization is to enhance detectability. It is performed either before (pre-column) or after (post-column) chromatographic separation of the amino acids. Among the most common labelling reagents used for post- and pre-column derivatization are ninhydrin and o-phthaldialdehyde (OPA), 6-aminoquinolyl-N-hydroxysuccinimidyl carbamate (AQC), phenylisothiocyanate (PITC) and 9-fluorenylmethyl chloroformate (FMOC) respectively [160]. However, there was a report of using OPA as pre-column derivatization to enhance sensitivity for fluorescent detection [137]. Derivatization process was made easier and simpler when using AQC as it is stable at room temperature for several days, has low toxicity, fewer side reactions and takes a shorter time to complete [143]. The use of other new derivatizing reagents are continually being reported and the main aim is to find a reagent that is fast reacted, highly sensitive and preferably stable at room temperature. Ethylchloroformate (ECF) and trifluoroacetylacetone (FAA) derivatization mixture gave better sensitivity and separation compared to using ECF alone [144]. A combination of ethyl chloroformate and ethanol highly separated enantiomer amino acids derivatives in foods using a chiral stationary phase GC [146]. The application of 4-chloro-7-nitro-2,1,3-benzoxadiazole (NBD-Cl) pre-column derivatization for CE-UV was successfully attained. NBD-CI produces a low number of by-products for fluorescent labelling of amino acids and UV detection and the cost is relatively cheap [149].

HPLC-FLD is a common method used to analyze amino acids in foods [137]. Although the proposed optimized method showed high sensitivity, reproducibility and rapid analysis of amino acids, showing potential for routine analysis, HPLC has also been coupled to other types of detectors. HPLC-DAD coupled with quadrupole time-of-flight mass spectrometry (QTOF-MS) and a chiral column were recently used to detect amino acids enantiomers in tea infusion [138]. This technique was fast and helps to identify the stages of amino acids enantioisomerization occurring in food samples. Dong and co-workers described the used of HPLC-MS/MS with a multiple reaction monitoring (MRM) mode [139]. The mobile phase, acetonitrile and water with 0.1% formic acid used in this method, help improved the separation of the amino acids and 5′-nucleotides, achieving good resolution and symmetric peak shapes for all analytes. The MS/MS and MRM mode may increase sensitivity, reproducibility, broad dynamic range, reduced analysis time and increased the throughput of the assay [161].

Lately, the use of UHPLC to analyze amino acids has become increasingly important, replacing HPLC due to its faster mode, higher reliability and reproducibility. The use of UHPLC and PDA to analyze amino acids in beer was reported by Redruello et al. [140]. This method allows more samples to be assayed per unit time, used less solvent, therefore reducing costs and associated waste and could be a potentially used for monitoring the safety and quality of beers or other similar beverages. Moreno-Rojas et al. [141] described the use of UHPLC-PDA coupled to high-resolution mass spectrometry (HRMS) to detect amino acids in shallots and black onions. In this method, the use of mobile phases; acetonitrile and acidified deionized water with ammonium acetate and ammonium formate gave better separation of the isomers Leu and Ile. The MS signal and peak shape were increased with the use of ammonium salts without affecting the sensitivity of the MS detector. HRMS optimized the gradient with the best resolution, shortest run time, increased accuracy and showed higher separation of twenty-one amino acids in the samples. A study using UHPLC-hydrophilic liquid chromatography (HILIC) in positive electrospray ionization (ESI) mode coupled with MS/MS (UHPLC-HILIC-ESI MS/MS) system to detect isobaric amino acids in cereal with a shorter run time was achieved using a tandem MS detection compared to using single MS detection [142]. A combined method of modified AQC and UHPLC-MS/MSto analyze amino acids in tea was developed with an improved detection sensitivity and resolution by increasing the separation of co-eluting compounds and shortening the chromatographic run time [143].

GC has long been developed and established for amino acids analysis in various food samples. The application of GC to detect amino acids in food matrices require the hydrolyzing of the samples followed by derivatization. An alternative method using GC-MS can be used to detect amino acids and their enantiomers especially when the volume of samples are very limited but higher sensitivity, resolution and speed are required [145,146,147].

CE has been a very useful technique for various food analysis including amino acids [162]. The development of CE methods for amino acids analysis mainly used UV detector and fused silica capillary column either with derivatized or underivatized amino acids. In order to increase efficiency and sensitivity, CE-UV is coupled with an online sweeping technology [150,151]. In this method, copper was used to enhancing the UV absorption by forming complexes [Cu (AA)n]^+2^ that has a stronger UV absorption. Another type of CE is capillary isotachophoresis (CITP) coupled with a conductometric detector (CD) [148]. The proposed method analyzed amino acids in cheese without derivatization which was much simpler compared to LC and results were acquired within a short time.

## 7. Fatty Acids and Cholesterol

Fatty acid (FA), an important component of lipid is commonly categorized as three major groups depending on the level of unsaturation; polyunsaturated fatty acids (PUFA), monounsaturated fatty acids (MUFA) or saturated fatty acids (SFA). The role of these dietary FA in human health defined by the different chain length as well as the number and position of double bonds [163,164]. Fatty acid composition determination in food is essential as it largely required for nutrition labelling, food research, product development, quality control, and physicochemical properties of a product [165]. Cholesterol, another key component in lipid is mostly found in animal origin products such as red meat, poultry, milk, eggs, yoghurt and cheese. Evaluation of cholesterol in food is crucial as dietary cholesterol associated with food sources of saturated fat which linked to health risk [166]. In addition, the cholesterol concentration information in food labeling allows consumer to monitor their cholesterol intake in their daily dietary intake and assist them in selection of healthy food.

### Sample preparation and Analytical Technique

Analysis of FA in food matrices involves several preparatory steps such as lipid extraction, derivatization process followed by identification and determination through different methods [167]. The determination of FAs in foods is most often carried out by GC based on the conversion of FAs into fatty acid methyl esters (FAME). Meanwhile, free FA (FFA) can be quantitated or detected after the conversion to FAME or directly as FFA after extraction from the food matrix.

There are many different approaches employed for the FA extraction from food matrices. The most common was the conventional liquid-liquid extraction using different organic solvent systems and solid-phase microextraction. While the derivatization process of FAs involves acid and base catalysis. Official methods such as AOAC 989.05 [168] and AOAC 969.33 [169] were adopted last few years for lipid extraction and preparation of FAME. Nevertheless, the conventional method has been improved over the years to suit the current technology, types of food matrix, cost as well as speed and less hazardous. Most recent, Manion et al. [170], has developed a novel butyl ester method which overcomes limitation of direct on-column injection and FAME methods. While another study employed frozen extraction method coupled with 2,4-dimethoxy-6-piperazin-1-yl pyrimidine (DMPP) derivatization which enhanced the selectivity and sensitivity for free fatty acid analysis in milk [142]. Contrarily, Kokotou et al. [171] managed to develop a direct quantification method of fatty acids in royal jelly without derivatization method. Another sample preparation improvement in meat sample was developed by Agnew and co-workers [172]. They have optimized conditions for one-step transmethylation by manipulating solvent concentration; incubation temperature and mixing mode using freeze-dried meat which offer an advantage for analyzing a broad range of fatty acids in various meat samples. Liu et al. [165] compared two methylation methods to evaluate fatty acids profile in milk. Other recent improvements in sample preparation for fatty acid analysis are listed in Table 6.

In addition to proper sample preparation, selection of appropriate analytical tool is paramount for fatty acid analysis. Wide range of analytical tools, including GC [173,174,176,179,182], GC-MS, gas-chromatography vacuum ultraviolet spectroscopy (GC-Vacuum UV) [178], gas-chromatography coupled with flame ionization detection (GC-FID) [170,172], liquid Chromatography-High Resolution Mass Spectrometry (LC-HRMS) [171,183], UHPLC-MS/MS [142], Raman spectroscopy [181], FTIR [188,189] and many more improved platforms have been used to perform FA analysis. Fatty acid analysis in LC-MS instrument showed some drawbacks in term of larger solvent consumption and lower selectivity [190]. However, a new platform such as the LC-HRMS method capable of direct determination of FA following simple sample preparation [183]. GC–MS has more advantages over GC-FID as the former could provide structural information, better separation and identification of FA isomers, supported with well-established databases for FAs identification with higher efficiency and better selectivity [191]. Raman spectra have significant advantage with aqueous systems as it is unobtrusive and able to detect grouped FA, such as total SFA, MUFA, PUFA, conjugated linoleic acid (CLA) and others. Qiu et al. [181] quantitated FAs in olive oil without any pre-treatment of the sample using Raman spectroscopy. While Liu et al. [165] combined GC-MS and LC-MS to analyze fatty acids in milk. Application of vacuum ultraviolet (VUV) detector in GC offers additional resolution and advantage of differentiating cis/trans isomers with niche selectivity for FAME analysis [178].

Sample preparation of cholesterol involves saponification, multistage solvent extraction followed by purification and concentration. Determination of cholesterol level can be divided into three major techniques: 1) classical chemical methods 2) fluorometric and colorimetric enzymatic assays 3) analytical instrument [192]. The official GC method used for cholesterol determination in foods is the AOAC 994.10 [193]. However, due to several limitations with the standard method, many alternative methods were developed to measure cholesterol in food matrices. Gema and Manuel [185] developed a quick and simple method to quantitate cholesterol in egg yolk using UV-VIS NIR spectroscopy. While Anna and co-worker [194], established an enzymatic method for total cholesterol in milk which is less time consuming, cost effective and suitable for large scale study. Another simple and robust quantitative method was suggested by Adu et al. [187] using the Liebermann-Burchard colorimetric reaction. The proposed methods could be an alternative option over the current employed method to quantitate cholesterol in food matrices. Table 6 highlighted several new technologies and analytical methods developed to evaluate the selected lipids which are essential components in the food composition.

## 8. Challenges in Generating Data for Food Composition Database

There are numerous challenges facing by analysts in producing reliable data for FCD. Artificial differences can be introduced to the data like data quality, sampling, sample processing/sample preparation and extraction, analytical methods, calculation methods or data expression. Cost in getting reliable analytical data is tremendously high especially the initial cost to purchase the instruments. However, the initial investment in instruments usually compensate by extensive number of samples that can be analyzed in shorter time as compared to the traditional method. Another cost is on consumables, chemicals and reagents for sample processing like freeze drying, sample preparation, extraction and analysis. There are also cost in participating quality control assurance program in ensuring the quality of the results is on-par with other laboratories participated in the program.

Food has significantly varied nutrient contents due to many reasons (i) environmental influences the feed, soil and climate, (ii) genetic resources of the food such as varieties, cultivar/breeds and processing of the samples like storage condition and fortification (iii) consumption pattern resulting in different recipe, brand names and fortification for the same food and (iv) food biodiversity or differences among products from different producers, (v) recipe formulation and (vi) cooking methods [4].

There are three approaches use by food composition data compiler in getting data for their FCD (Figure 1). The first approach which is most reliable and expensive is using the original analytical data. However, some compilers also using data from recipe calculation especially for cooked foods due to the abundance and increasing variety of these kinds of foods. It is impossible to analyze nutrients of all cooked dishes, as this would considerably raise the cost of food analysis for a comprehensive FCD. Nevertheless, they are many different recipe calculation procedures available and factors used vary between different countries. A standardized procedure for the management of cooked foods in FCDs is desirable to enable between country comparisons. Data from published articles and food labelling from food producers can also been used for FCD however, a systematic and standardized approach is needed to evaluate the quality of the published data before using it for FCD.

Ruth Charrondiere et al. [195] has emphasized three pillars which are needed to ensure high-quality food composition data are generated, compiled, disseminated and used. Firstly, international standards, guidelines and tools on the generation and compilation of food composition data must be developed and used. Secondly, national and/or regional food composition programs must exist, which are updated regularly, and thirdly, human resource must be trained in all aspects related to food composition.

## 9. Conclusions

Food analysis is very important for FCD by providing reliable data for the use in many areas of nutrition, food science and health. The scope of both sample pre-treatment and analytical method development is a broad topic that involves continuous exploration of various approaches and improvement in order to be in line with current requirements. Hence, it is important that new chemistries along with enhanced detection possibilities and improved sample preparation for various matrices are continually ventured. We have highlighted several new methods which pave the way to overcome limitations in the conventional methods for various nutrients. Moreover, the application of new technology involves mild sample preparation, avoids time-consuming derivatization, and allows direct quantification with a large sample size which provides reliable results in agreement with conventional methods. Besides that, most methods were simplified thus allows the processing of a higher number of samples, shorter assay, at the same time, minimizing the sample manipulation and errors linked to analytical method, consequently, the sample loss and contamination. This outlook is believed to be capable of generating a standardized approach of the analytical method for accurate and reliable determination.

## Figures and Tables

**Table 1:** Methods, food matrices and advantages of current improved technique for proximate.

Sample Preparation	Instrument	Food Matrix	Advantages of Current Improved Technique	Ref.
**Moisture**				
Heating of sample through absorption of IR radiation from a halogen radiator. Continual determination of mass during drying process. The moisture content percentage is determined from the difference in weight before and after drying.	Halogen moisture analysers	All type of food matrix	Highly energy-efficient, less water-consuming, and environmentally friendly compared to conventional heating. Further, it is also characterized by homogeneity of heating, high heat transfer rate, low heating time, low energy consumption, improved product quality, and food safety	[9]
Weighed and spread the sample as thin layer in a Petri dish before placing on the circular asbestos sheet nearby the center. Heated at different watts of absorbed microwave heating (MW) power output settings. Prior to obtaining the weight, MW dried sample was stored in a desiccator containing silica gel to decrease the surface moisture and high temperature development in the sample. The weight loss after each MW drying was expressed as the apparent moisture content (m.c.) of the samples.	Microwave oven	Paddy varieties	An extremely rapid method of drying up a sample but the temperatures achieved is very high, making it suitable only for very thermostable materials. It is also not useful if the moisture content is below 2%.	[10]
Samples were evenly spread in a glass Petri dish. A circular black paper was placed at the bottom of the Petri dish under the samples to avoid specular reflections from the bottom of the dish. The Petri dish was placed on the turntable, the turn table was set in motion and the reflectance spectrum was recorded while scanning the sample along a wide periphery within the Petri dish. Reflectance spectra were collected in the wave-length range between 1000 nm and 1800 nm, at 1 nm intervals. An integration time of 10 ms was used throughout the measurements	NIR	Cereal grains	Demonstrated reliable prediction of wheat composition directly on the whole kernels, which represented a great advance with benefits in terms of sample preparation, cost, and applicability	[11]
The experiments were carried out with two unilateral magnets. One of the magnets (denoted as magnet A). The other magnet (magnet B) has a non-linear magnetic field. Magnet B features a high signal-to-noise ratio (SNR) due to a large sensitive spot. Both magnets were integrated with a Bruker Minispec console. The samples were packed into 1 × 1 × 4.5 cm plastic prism containers, chosen to fit the linear gradient region of magnet A for testing the diffusion-weighted methodology.	NMR	Beverages, oils and lipids, vegetables, meat, and dairy products	A robust method, which can rapidly analyse mixtures at the molecular level without requiring separation and/or purification steps	[12]
**Total Protein**				
A sample of known mass is combusted in a high temperature (about 900 °C) chamber in the presence of oxygen. This leads to the release of carbon dioxide, water, and nitrogen. The nitrogen content is then measured by passing the remaining gasses through a column that has a thermal conductivity detector at the end. Thus, the signal from the thermal conductivity detector can be converted into nitrogen content.	Enhanced Dumas method	All food matrix	It is much faster than the Kjeldahl method (under 4 min per measurement, compared to 1–2 h for Kjeldahl). It doesn’t need toxic chemicals or catalysts. Many samples can be measured automatically. It is easy to use.	[13]
**Total Fat**				
Liquid-phase MAE process is based upon the ability of a matrix to absorb microwave energy.	MAE	Cheese	MAE offers a range of benefits over other solvent extraction methods, since MAE is faster and more effective, has lower consumption of energy and solvents, and, above all, uses less toxic solvents. It performs two steps in only a single step, i.e., hydrolysis and extraction simultaneously	[14]
**Total Dietary Fibre (TDF)**				
RITDF method combines the key attributes of AOAC Official Methods 2002.02 [15], 985.29 [16], 991.43 [17], 2001.03 [18] and 2009.01 [19].	Integrated Total Dietary Fiber Assay Kit	All food matrices	RITDF method is more accurate because it is specifically designed to overcome both potential inaccuracies: the double measurement of some fibres and the lack of measurement of other fibres. Since RITDF method improves the accuracy of fibre analysis, the determination of available carbohydrate will also be more accurate. This test may replace the need for multiple tests, highlights possibility for potential savings.	[20]
**Ash**				
Depends on sample preparation and measurement method. First—the samples were covered with a slide window and clamped to the ATR diamond crystal using pressure gauges; second—the samples were placed on the ATR diamond crystal and clamped using pressure gauges. The tight fit of the ATR clamp head shape to the gap above the crystal allowed an accurate and even coverage of the crystal with a thin layer of the sample.	ATR-FTIR	Vegetable/Plant	In general, the proposed method requires a small drop/amount of sample on the ATR base-plate reagent consumption, being much faster than traditional techniques, allowing potential applications for simultaneous determination of sulphur, nitrogen and ash contents for routine analysis of plant/vegetable tannins by FTIR data.	[21]
**Total Sugar**				
Date syrup was dried before dissolving in pyridine and placed in an ultrasonic bath. The sample was mixed well by vortex and centrifuged to remove any insoluble materials. Part of the supernatant was taken for the oximation-silylation step.	GC-MS	Date juice (possibility of other food matrices)	Rapid sugar identification GCMS results determined the appropriate enzymatic assays for quantifying the sugars in date juice. These results were similar to those of the two enzymatic methods (standard enzymatic assay and measuring the change in pH by CL10 analyser).	[22]

**Table 2:** Methods, food matrices and advantages of current improved technique for minerals.

Sample Preparation	Instrument	Application/Food Matrix	Advantages of Current Improved Technique	Ref.
**Open Digestion** Open vessel hot block digestion uses atmospheric pressure digestion for extraction of high throughput samples [33]. **Microwave** **1. Microwave assisted-digestion (MW-AD)** **2. Microwave induced-combustion (MIC)** **Slurry Sampling** A technique of direct sample preparation applicable only for Graphite Furnace AAS.	Flame & Graphite Furnace AAS	Brown sugar, wine, fruit juice, honey, meat and baby foods, chocolate, vegetables, infant formula, fish fillet, vegetable oil	Open digestion offers high throughput, AAS provides high sensitivity, good precision, low cost, relative simplicity	[35,36,37,38,39,40,41,42]
	ICP-OES/AES	Almonds kernel, tea leaves, coffee, cereals, mussel tissues, mushrooms, seafood, cow’s milk, legumes, wine, nuts, cheese, onion, garlic, honey, barley, bread, fish, sugarcane juice, soft drinks	MW-AD able to digest difficult food samples matrix quickly, completely, minimum loss of volatile compounds and reduces risk of contamination. MIC uses diluted solutions and lower reagent comsumption accordance with green chemistry recommendations. Slurry Sampling capable minimising drawbacks of manual and automated sample digestion. ICP-OES/AES provides rapid elemental determination techniques, Multiple elements can be analysed from small volume of samples, Refractory samples that are lower concentration can also be determined, By using plasma source, non-metals can be determined	[43,44,45,46,47,48,49,50]
	ICP-MS	Meat, honeydew honey, vegetables, milk, rice, spices and aromatic herbs. maize	High sensitivity for trace element detection, multi-elemental and isotopic analysis, and high sample throughput.	[51,52,53,54,55,56,57,58,59]
Dried samples powder was pressed until the surface was homogenous and ready for analysis.	ED-XRF	Fruits and vegetables, cumin spice	Simple sample preparation, direct measurement, Multi-element analysis, fast analysis.	[45,60]

**Table 3:** Methods, food matrices and advantages of current improved technique for fat soluble vitamins and carotenoids.

Sample Preparation	Instrument	Food Matrix	Advantages of Current Improved Technique	Ref.
**Vitamin A**				
Deproteinization with ethanol followed by direct hexane extraction	HPLC-DAD-MS/MS-APCI (+)-NARP	Cow, buffalo, goat and ewe’s milk	Novel analytical method with increased selectivity, sensitivity for characterization of retinoic acid, retinal, retinol and fourteen retinyl esters.	[83]
**Vitamin D**				
Graphene-coated magnetic particle (Fe_3_O_4_@Graphene based Magnetic Solid-Phase Extraction (MSPE)	HPLC-UV	Milk	Reduced time, lower consumption of organic solvent, improved sensitivity and accuracy, eliminates the need for protein removal prior to extraction of vitamin D (D_2_, D_3_).	[84]
Magnetic three-dimensional graphene-sporopollenin sorbent (3DG-Fe_3_O_4_@Sp) based dispersive micro-solid phase extraction (MD-μ-SPE)	HPLC-UV	Bovine milk	New sorbent material (3DG-Fe_3_O_4_@Sp) is synthesised and applied for the extraction of vitamin D_3_. Proposed technique is advantageous for its low solvent consumption, low sorbent dose, as well as rapid extraction and analysis.	[85]
Ultrasound assisted extraction (UAE) followed by DLLME	HPLC-UV	Wheat flour, bread	Accurate, precise, reliable sample pre-treatment method with reduced sample-matrix interference and good detection limit for trace levels of vitamin D_3_.	[86]
**Vitamin E**				
Overnight cold saponification followed by LLE with hexane	HPLC-APCI (+)-MS/MS-isocractic NARP	Pecan nuts	Simultaneous quantification of four tocopherols and tocotrienols, each with a highly sensitive method that explains detection of minor homologues (δ-tocopherols and tocotrienols) for the first time.	[87]
Extraction into silicone oil, acting as lipophilic binder of GCPE	ED by SWASV	Margarines and edible oils	Results obtained are comparable to HPLC, with respect to total tocopherol content. Electrochemical approach provides an option for easy sample preparation, rapid and cheaper analysis.	[88]
**Vitamin K**				
Overnight cold saponification followed by LLE with hexane	HPLC-APCI (+)-MS/MS-NARP	Human milk	Simultaneous detection of phylloquinone (vitamin K_1_), menaquinone-4 (MK-4) and menaquinone-7 (MK-7) in human milk with high accuracy and precision. Utilizes fewer samples with a simplified and inexpensive extraction procedure.	[80]
ASE system followed by extract clean-up via SPE	LC-APCI-MS/MS	Fruits and vegetables	Combination of ASE and LC-APCI-MS/MS technique provides a sensitive, selective and rapid approach for vitamin K_1_ analysis in fruits and vegetables.	[81]
Ultrasonic assisted solvent extraction and SPE	UHPLC-APCI (+)-MS/MS	Fermented foods	Minimal use of chlorinated solvents and columns with smaller core shell particles dimension enable lower flow rate with good resolution. Post-column derivatization is eliminated with the use of tandem-MS and results in better detection limits. Proposed technique offers excellent selectivity, sensitivity and rapid analysis of phylloquinone and menaquinones.	[89]
Adsorptive accumulation onto solid GCE surface	ED by (SWAdSV)	Extra virgin olive oil	Electrochemical approach offers benefits of lower solvent consumption, easier sample preparation as well as lower cost. Better analytical performance is also seen in comparison to other electroanalytical methods. Results obtained are comparable with HPLC technique.	[90]
**Simultaneous Analysis of Selected Vitamins**				
Protease digestion	HPLC-dual wavelength FLD and DAD	Infant formulae and fortified milk powders	Simultaneous detection of vitamin A, E esters and β-carotene. Method eliminates saponification that allows other esters to be used as an internal standard. Faster extraction with adequate precision and recovery using protease digestion.	[91]
Direct solvent extraction using enzyme-assisted matrix disintegration and methanolic protein precipitation	SFC-APCI (+)-MS/MS	Milk-based infant formula, infant cereals, adult nutritionals, frozen mixed meals	Simultaneous analysis of vitamin A (retinyl acetate, palmitate, retinol), vitamin E (α-tocopherol, α-tocopheryl acetate), vitamin K (phylloquinone, menaquinone-4) via direct injection and vitamin D (D_2_, D_3_) upon derivatization. Fast, easy, robust, safe, lower cost and reliable technique for all four FSV analyses.	[92]
Simplified saponification and solvent extraction	UHPLC-DAD with on-line SPE	Cereal and flour products	Simultaneous analysis of vitamin D, K (D_2_, D_3_, K_1_, K_2_) with on-line SPE application for further sample purification and better detection at trace levels. Simple and reliable UHPLC method of high accuracy, repeatability and recovery.	[93]
DSPE with newly synthesized polymeric material consisting of PAA-T	HPLC-DAD	Milk and egg yolk	Simple, cost-effective, sensitive technique for simultaneous determination of vitamin A and E. First reported application of polyacyril amide and tetracycline as solid phase sorbent; where the use of tetracycline is useful as it is of low cost and provides good zones for the interaction of vitamin molecules.	[82]
Dilution with MTBE, sonication, filtration	UPC^2^-PDA	Canola, sunflower, vegetable, mixed, and coconut oil	Rapid detection of seven FSV (retinol, retinyl acetate, D_2_, D_3_, α-tocopherol, K_1_, K_2_) and carotenoids (lutein, lycopene, β-carotene) within 8 and 3 min, respectively. UPC^2^ technique environmental-friendly, cost-effective, with improved repeatability and faster analysis compared to HPLC.	[74]

**Table 4:** Methods, food matrices and advantages of current improved technique for water-soluble vitamins.

Sample Preparation	Instrument	Food Matrix	Advantages of Current Improved Technique	Ref.
**Vitamin C**				
Addition of MPA, centrifugation, reduction to DHAA	HPLC-DAD	Juices, fruits, vegetables, fruit cream powder and infant milk formula	Selective and precise method for determination of vitamin C in foods.	[98]
Addition of MPA, centrifugation, dilution	UPLC-PDA and HPLC-PDA	Fruit beverages	UPLC method is faster, more sensitive, consumes less eluent, cheaper and more eco-friendly than the conventional HPLC method.	[99]
PLE, acid extraction and maceration	UHPLC-DAD	Camu-came fruit	PLE technique give extracts rich in vitamin C and using nontoxic solvents. Fast, higher resolution, greater sensitivity and specificity for determination of L-AA and DHAA.	[100]
Homogenized, Addition of EDTA, centrifugation, dilution	LC-MS	Fruits (apple, kiwi and orange)	Higher sensitivity and selectivity for determination of the L-AA and DHAA.	[101]
Addition of MPA, centrifugation, filtration, derivatisation	Voltammetric trace analyser 746 VA	Juices, fruits and vegetables, fruit cream powder and infant milk formula	High selectivity, lower costs, shorter time and simple method for determination of total vitamin C and DHAA contents in food.	[102]
Liquid extraction	UV-Visible Spectrophotometer	Fruits	Simple and fast method for determination of AA.	[103]
**Thiamine (Vitamin B_1_)**				
PVPP pre-treatment and derivatization	HPLC-FLD	Red wines	Higher recoveries and accurate method for determination of thiamine vitamers (thiamine diphosphate, thiamine monophosphate and thiamine) in wines.	[104]
Protein precipitation, enzymatic treatment	UPLC-FLD	Milk	Simple, fast, cost effective UHPLC method for the determination of the three most relevant vitamin B_1_ active compounds, namely thiamine, thiamine monophosphate and thiamine diphosphate.	[105]
** Riboflavin (Vitamin B_2_) **				
Centrifugal skimming, ultrafiltration	HPLC-FLD	Milk and milk products	Reliable and accurate method without strong acidic conditions for determination of riboflavin and the related flavins (flavin mononucleotide and flavin adenine dinucleotide).	[106]
** Niacin (Vitamin B_3_) **				
Dilution and derivatization	MCE-LIF	Functional Drink	Rapid, low sample consumption, miniaturization and high sensitivity.	[107]
Acid treatment, protein precipitation, filtration	HPLC-FLD	Meat, cereal and legume	Accurate method for determination of vitamin B_3_ (nicotinic acid and nicotinamide) profiles in animal and plant-based foods.	[108]
** Pyridoxine (Vitamin B_6_) **				
Acid digestion, enzyme treatment	HPLC-ED	Cereals products	Simple, fast sample preparation, sensitivity and selective method for simultaneous analysis of three vitamin B_6_ vitamers (pyridoxamine, pyridoxal and pyridoxine).	[109]
** Biotin (Vitamin B_7_) **				
Acid treatment	HPLC-UV	Milk	Rapid, selective, reproducible and high adsorption capacity for determination of biotin in milk food samples.	[110]
** Folates (Vitamin B_9_) **				
Buffer extraction, enzymatic treatment, filtration	UFLC-DAD	White rice	Fast and good recovery method for analysis of folic acid in white rice.	[111]
Enzyme treatment, SPE	LC-MS/MS	Dairy products, cereals, legumes, fruit, vegetables, offal and meat	Rapid, sensitive and reproducible method for analysis of six folates in food.	[112]
** Cobalamins (Vitamin B_12_) **				
Protein precipitation, SPE	LC-MS/MS	Cow’s milk	Fast and better selectivity for determination of vitamin B_12_ homologues.	[113]
Enzymatic treatment, centrifugation, filtration, purification	HPLC-DAD	Vegetables and fruits	Good selectivity, recovery and repeatability for the accurate determination of vitamin B_12_ in complex matrices.	[114]
** Simultaneous Method of Water-Soluble Vitamins **				
Filtration, Degassing	HPLC-DAD	Functional beverages	Fast, high accuracy and good reproducibility for determination of seven WSVs (vitamin C (AA), vitamins B_6_, B_2_, B_3_ (nicotinamide and nicotinic acid), B_9_ and B_12_) in two functional beverages.	[115]
Filtration, d-SPE	HPLC-UV	Orange Juice	Less consumption of organic solvents. High selectivity and satisfactory recovery for determination of vitamins B_2_, B_3_ and B_6_ in juice.	[116]
Sonication, protein precipitation, extraction with diethyl ether	LC-MS	Fresh Milk	Low volume of samples and simple sample preparation. Highly sensitive methods to quantify vitamins B_1_, B_2_, B_3_, B_5_, B_6_, B_7_ and B_9_ from milk samples.	[117]
Acid hydrolysis, acidic hydrolysis plus peptide precipitation, acidic plus enzymatic hydrolysis and enzymatic hydrolysis	LC-MS/MS	Anchovies	Fast and high specificity for simultaneous quantification of riboflavin, nicotinamide and nicotinic acid in anchovies.	[118]
Centrifugation	Autolab with PGSTAT 302N	Fruit Juices and energy drinks	Fast, simple, selective and sensitive method for determination of AA and vitamin B_6_ in fruit juices and energy drinks.	[119]
Degassing, centrifugation, addition of MPA, filtration	MEKC-UV	Energy drink, sport drink and fruit nectars	Minimal sample preparation and reagent consumption. Simultaneous determination of eight WSVs (vitamins B_1_, B_2_, B_3_ (nicotinamide and nicotinic acid), B_5_, B_6_, B_12_ and C).	[120]

**Table 5:** Methods, food matrices and advantages of current improved technique for amino acids.

Sample Preparation	Instrument	Food Matrix	Advantages of Current Improved Technique	Ref.
Sonicated samples and pre-column derivatization using *O*-phthalaldehyde (OPA).	HPLC-FLD	Vegetables and commercial juices	First reported OPA derivatives to analyse amino acids using C8 column. A rapid, sensitive, accurate and reproducible method for simultaneous determination of twenty-one amino acids (Asp, Glu, Asn, His, Ser, Gln, Cit, Arg, Gly, Thr, Ala, β-ala, Tyr, Met, Val, Trp, Phe, Ile, Leu, Lys), including non-proteinogenic amino acid, Orn.	[137]
SPE-concentrated samples without derivatization except for the analysis of DL-theanine, samples were pre-column derivatized using AccQ-Tag reagents.	HPLC-DAD-QTOF-MS (Chiral)	Tea	Rapid sample preparation (underivatized) and sensitive method for the detection of eleven types of D-amino acids (Thea, Thr, Leu/Ile, Phe, and Tyr) including L-form of theanine in tea infusion.	[138]
Deproteinization of samples with ice-cold methanol at 4 °C for 10 min, underivatized.	HPLC-MS/MS	Shitake mushroom	The use of LC-MS/MS eliminates derivatization step and allows for overlapping amino acid retention times, shortening the analysis time of determining simultaneously twenty amino acids (Pro, Thr, Cys, Asn, Lys, Met, Phe, Arg, Asp, His, Gly, Glu, Ala, Ile, Leu, Ser, Trp, Tyr, Val, Gln) and six 5′-nucleotides using a C18 column. Ion-pairing reagent, acetonitrile and water with 0.1% formic acid were shown to improve the separation of amino acids and 5′-nucleotides, achieving good resolution and symmetric peak shapes for all analytes.	[139]
Derivatization using diethylethoxymethylenemalonate (DEEMM).	UHPLC-PDA	Beverage (Beer)	Rapid analysis, high sensitivity and reproducibility. Used less solvent and can be a potential routine analysis for safety and quality of beers or other similar beverages. A novel UHPC method using a C18 column for a simultaneous determination of twenty-one amino acids (Asp, Glu, Asn, Ser, Gln, His, Gly, Thr, Arg, Ala, Pro, Tyr, Val, Met, Trp, Ile, Leu, Lys and Phe) including Orn and GABA, 9 biogenic amines and ammonium ions in beer.	[140]
Hydrolysed samples with a mixture of deionized water and methanol (20:80, *v*/*v*) acidified with 1% formic acid, underivatized.	UHPLC-PDA-HRMS	Vegetables (Fresh shallot and black onions)	Better separation of Leu and Ile isomers. Ammonium salts increased the MS chromatogram signal and peak. Simultaneous detection of twenty-one amino acids (Leu, Ile, Phe, Trp, Met, Val, Pro, Tyr, Ala, Thr, Gly, Glu, Gln, Ser, Asn, Lys, His, Asp, Arg, Orn and GABA), using BEH amide column (HILIC). Potential applicability to other similar vegetables.	[141]
Hydrolysed samples with 6M HCL with reducing agent, 4% (*v*/*v*) thioglycolic acid, underivatized.	UHPLC-HILIC-MS/MS	Cereal (Wheat flour)	The use of HILIC column enhanced the sensitivity of electrospray ionization-mass spectrometry (ESI-MS) detection. Tandem MS increases resolution and decreases run time, shorter separation time, high resolution and sensitive for a simultaneous determination of seventeen amino acids (Gly, Ala, Ser, Pro, Val, Thr, Asp, Glu, Ile, Leu, Asn, Lys, Met, His, Phe, Arg, Tyr and Cys).	[142]
Samples extraction using water (30 min), pre-column derivatization using 6-Aminoquinolyl-*N* hydroxysuccinimidyl carbamate (AQC) (AccQ-Tag reagent).	UHPLC-TQ-MS/MS	Tea	Simple extraction method. AQC reduced derivatization time, stabile at room temperature for several days, low toxicity, simple derivatization process and fewer side reactions. TQ-MS/MS improved detection sensitivity and resolution, increasing the separation of co-eluting compounds and shorten the chromatographic run time for simultaneous detection of twenty-one free amino acids (Asp, Glu, Hy-pro, Ser, Gly, His, Thr, Ala, Arg, Pro, Thea, Cys, Tyr, Val, Met, Ile, Lys, Leu, Phe, Trp and GABA) using a C18 column.	[143]
Derivatization using Trifluoroacetylacetone (FAA) and Ethylchloroformate (ECF).	GC-MS	Jams, fruits and pharmaceutical preparations	Two-stage derivatization with FAA and ECF in an aqueous phase showed better sensitivity and selectivity. This method simultaneously analysed nineteen amino acids (Gly, Ala, Val, Leu, Ile, His, Ser, Thr, Cys, Met, Asp, Asn, Pro, Glu, Gln, Lys, Tyr, Trp and Phe) using HP-5 column.	[144]
Hydrolysed samples with 0.1M HCL, deproteinised with acetonitrile and derivatization with N-methyl-N-(tert-butyldimethylsilyl trifluoroacetamide (MTBSTFA).	GC-MS	Meat (Dry-cured ham and Fresh pork loin)	Lesser time, a lower amount of sample and solvent required, cost and time-effective. Good recovery and excellent linearity except for Trp. Simultaneous detection of twenty-one amino acids (Ala, Gly, Val, Leu, Ile, Pro, Met, Ser, Thr, Phe, Asp, Cys, Glu, Asp, Lys, Gln, Arg, His, Tyr and Trp) including Hydroxyproline.	[145]
Derivatization with ethyl chloroformate/ethanol mixture.	GC-MS (Chiral)	Kefir (Fermented milk)	Combination of ethyl chloroformate and ethanol was found to be the best derivatization reagent to separate and quantify a higher number of enantiomer amino acid derivatives. This method successfully detected d- and l-ala, d- and l-val, d-pro, l-thr, Asp and Glu, Met and Cys.	[146]
Derivatization consisted of solid-phase extraction clean up and using reagent alkyl chloroformate.	GC-MS	Honeydew honey	Alkyl chloroformate produced stable derivatives at room temperature. Fast analysis (7 min) and no matrix effects were detected in the studied range. The standard mix of thirty-two amino acids was successfully separated.	[147]
Hydrolysed samples with 0.1M HCl, triple extractions (30 min each process), underivatized.	CITP-CD	Cheese	Much simpler compared to LC due to the direct injection of samples without derivatization, high sensitivity and precision. CD and PTFE pre-separation capillary analysed six amino acids (His, Phe, Lys, Arg, Tyr and Orn) with short running time.	[148]
Hydrolysed samples using 80% (*v*/*v*) ethanol and pre-column derivatization with 4-chloro-7-nitro-2,1,3-benzoxadiazole (NBD-Cl).	CE-UV	Potato, eggplant, chickpeas, soft wheat flour and Sorghum Durra flour	NBD-CI is low cost and produces a low number of by-products for fluorescent labelling and UV detection using fused silica capillary column. Superior resolution and sensitivity compared to HPLC technique for the determination of six amino acids (Ala, Asp, Glu, Pro, Ser and Val) in various foods matrices.	[149]
Samples extraction using carbon tetrachloride, underivatized.	CE-UV (with online sweeping technique)	Beverage (Beer)	A novel method for the analysis of amino acids in beer without derivatization. The use of copper ions helps to enhance the UV absorption by forming complexes [Cu (AA)n]^+2^ that has a stronger absorption rate. An online sweeping technique enhanced the sensitivity of the amino acids, improved 25 ~ 35-fold. Fourteen amino acids (Lys, Gly, His, Ala, Ser, Val, Met, Phe, Leu, Ile, Try, Pro and Glu) were separated and determined.	[150]
Sample extraction using carbon tetrachloride, underivatized.	CE-UV (with online sweeping technique)	Fermented soy sauce	Higher resolution, shorter analysis time and sensitive compared to the LC technique. Fourteen amino acids (Lys, Gly, His, Ala, Ser, Thr, Val, Phe, Leu, Ile, Trp, Pro, Glu and Asp) were separated and determined using uncoated fused silica capillary column.	[151]

**Table 6:** Methods, food matrices and advantages of current improved technique for fatty acids and cholesterol.

Sample Preparation	Instrument	Food Matrix	Advantages of Current Improved Technique	Ref.
**Fatty acids**				
Combination of lipid extraction and derivatization with the base-catalyzed method followed by trimethylsilyl-diazomethane (TMS-DM)	GC	Margarines	This method was found to be effective tools for analyzing dietary fats and oils in complex mixtures of food products for monitoring of low levels of FA and TFA, and the control of labelling authenticity.	[173]
The first method for triglycerides analysis requires dissolution of the sample in n-hexane and GC analysis using a capillary column. The second method is based on the transesterification of triglycerides as pentyl esters in a single- step reaction using sodium pentanoate in pentanol. The third method involves the transesterification of triglycerides in fat through reaction with 2-phenylethanol in a single step	GC	Butter	The first method does not require the stabilization phase of the GC system that is required in the official method. It is a simple method based on the chromatogram overlap. The advantage of second method; using pentyl esters reduces the volatility of short-chain FAs, and substantial recoveries were obtained compared with methyl ester analysis. The third method allows LC analysis at room temperature without degradation	[174]
Total lipid was extracted by the Folch method. Three methods of transesterification were compared. Method 1: 0.2 M KOH in methanol at 50 °C for 20 min; Method 2: 6% H_2_SO_4_ in methanol at 60 °C for 2 h; Method 3:6% H_2_SO_4_ in methanol at 80 °C for 60 min.	GC-MS and LC-MS	Milk	This study proposed simple one-step protocol based on 0.2 M methanolic KOH, a short reaction time (20 min) and a mild reaction temperature (50 °C) for milk FAME preparation	[165]
Two solid-liquid extraction; Protocol A using methanol and Protocol B using diethyl ether/isopropanol	LC-HRMS	Royal Jelly	Using a new platform, LC/HRMS to analyze free fatty acids in royal jelly. Solid liquid extraction protocol gives good recoveries and the method allows fast and direct quantification of FFA besides offering an advantage to simultaneously screen for additional potential fatty acids in royal jelly without the need of reference compound.	[171]
FAMEs were prepared using in situ methylation according to [175] with extraction process	GC	Fermented milk	Direct methylation reduces time, solvent consumption, being cost efficient and environmentally friendly, whereas free of interferences due to solvent affinity. This method allows quantification of CLA with good reproducibility	[176]
FAME derivatization procedure using BF_3_ 14% in methanol according to International Standards—ISO 5509 (2000), indicated by the AOCS Method Ce 1j-07. The FAMEs in the fat samples were identified by the GLC procedure using the modified temperature program, by comparison of their relative retention times calculated to 18:0 with the respective relative retention times of the 52 FAMEs in the GLC-463 standard	Gas-Liquid Chromatography (GLC)	Mixture of soybean and sunflower oil, fish oil, butterfat	The proposed method allows to completely separate butyric acid from the solvent, trans-18:1 from cis-18:1, 20:1 isomers from 18:3n-3, 22:1n-9 from 20:4n-6, 20:5n-3 from 24:0 and the main CLA isomers	[177]
Oil samples were trans-esterified using 3 N methanolic HCl	GC-VUV	Olive oil, canola, vegetable, corn, sunflower and peanut oil	GC-VUV has niche selectivity and able to distinguish unsaturated FAMEs easily and differentiate cis/trans-isomeric FAMEs with enhanced chromatographic separation supported by deconvolution capabilities of the VUV detector and software	[178]
Fatty acid methyl ester (FAME) sample separation performed isothermally at 180 °C.	GC	Rapeseed oil mix	Ionic liquid (IL)-based column exhibited good selectivity in the analysis of the cis/trans C18:1 isomers of a partially hydrogenated vegetable oil sample on 30-m columns	[179]
Solid phase extraction and converted to fatty acid butyl esters (FABE)	GC-FID	Dairy products	FABE method overcomes limitations associated with direct on-column injection, such as either column-phase absorption or deterioration, accurate quantification of short-chain free fatty acids, and underestimation of polyunsaturated free fatty acid. This method is applicable for the quantification of FFAs in a wide range of dairy products	[170]
Frost pre-treatment of samples and coupled with an *n*-hexane extraction. The samples were further derivatized using 2,4-dimethoxy-6-piperazin-1-yl pyrimidine (DMPP)	UHPLC-ESI-MS/MS	Milk powder	Frozen pre-treatment of samples improved the extraction efficiency of FFAs in infant milk powder due to the increased polarity from water to ice while decreases impurities in the extracts. High sensitivity and specificity of DMPP labelling coupled with MS reduced sample amount.	[180]
Samples were directly used without any treatment	Raman Spectroscopy	Olive oil	Relative Raman intensity analysis useful for quick quality evaluation of extra virgin olive oils	[181]
Samples were extracted using acid and base catalyst method. Acid catalyst were carried out in H_2_SO_4_/methanol solvents. Methylation was performed for 2 h at 80 °C, and FA methyl esters were recovered for chromatographic analysis by the addition of isooctane. The extraction using base catalysts was conducted by AOAC 989.05 [168] official method, followed by AOAC 969.33 [169] for methylation.	GC	Milk products	Acid catalysts method is able to extract CLA and some fatty acids with higher yield compared to base catalyst. This method minimizes sample loss and contamination through reduced sample manipulation.	[182]
All meat samples were freeze dried and ground to fine powder. FA derivatized using bi-methylation procedure	GC-FID	Meat	This method has fewer steps and can be performed under non-anhydrous conditions. This method is also applicable to meat samples from different species, covering a broad range of fat content and offers a simplified and reliable method for analysis of fatty acids from meat samples.	[172]
Liquid/liquid extraction protocol involving the addition of methanol for the protein precipitation.	LC-HRMS	Milk	The current method involves mild sample preparation conditions, excluding the hydrolysis of esterified fatty acids of triacylglycerols or other lipid classes and avoids time-consuming extraction pre-separation, or derivatization procedures. It is rapid and robust, permitting the quantification of twenty-two FFAs in a 10-min single run	[183]
**Cholesterol**				
Hot saponification with subsequent derivatization to trimethylsilyl ether	GC	Turkey meat	An easy, quick and sensitive GC method for the determination of cholesterol in turkey meat products in the range of 0.4–8 mg cholesterol/g using relative response factors	[184]
Saponification at 80 °C for 3 h for a complete cholesterol extraction	UV-VIS-NIR spectroscopy and enzymatic method	Egg yolk	The UV-VIS-NIR spectroscopy combined with chemometric tools demonstrated to be a useful, rapid, clean and cheap technique for determination of egg yolk cholesterol	[185]
Saponification process with different amount of samples (0.1, 0.25, 0.5, 0.75 and 1.0 g, wide range of ethanolic KOH concentrations (0.1, 0.2, 0.3 and 0.4 M) and different saponification reaction time (30, 60, 90 and 120 min)	HPLC and UHPLC	Sour cream, egg, egg yolk and chicken nugget	UHPLC method allowed reduction in the consumption of organic solvents (8 times lower) and decreased analysis time (4 min), being more eco-friendly, than conventional HPLC methods	[186]
Saponification with 10% methanolic KOH and separated in a solvent-solvent extraction with diethyl ether:water (5:2)	Spectrophotometer	Dairy products	Liebermann-Burchard reaction via colorimetric method is a robust and reliable alternative method for analysis of cholesterol in dairy products	[187]

## Abbreviation

AA	Ascorbic acid
AAS	Atomic Absorption Spectrometer
Ala	Alanine
AOAC	Association of Official Analytical Chemists
APCI	Atmospheric pressure chemical ionization
AQC	6-aminoquinolyl-*N*-hydroxysuccinimidyl carbamate
Arg	Arginine
ASE	Accelerated solvent extraction
Asp	Aspartate
Asn	Asparagine
ATR-FTIR	Attenuated total reflection Fourier-transform infrared spectroscopy
BCAA	Branched-chain amino acids
β-Ala	β-alanine
CE	Capillary electrophoresis
CD	Conductometric detector
CITP	Capillary isotachophoresis
Cit	Citrulline
CLA	Conjugated linoleic acid
Cys	Cysteine
DAD	Diode array detector
DEEMM	diethylethoxymethylenemalonate
DHAA	Dehydroascorbic acid
DLLME	Dispersive liquid-liquid microextraction
DMPP	2,4-dimethoxy-6-piperazin-1-yl pyrimidine
DSPE	Dispersive solid-phase extraction
2D-LC	Two-dimensional liquid chromatography
EAA	Essential amino acid
ECF	Ethylchloroformate
ED	Electrochemical detector
ED-XRF	Energy-dispersive X-ray fluorescence
EDTA	Ethylenediaminetetraacetic acid
ELSD	Evaporative light scattering detector
ESI	Electrospray ionization
FA	Fatty acid
FAA	Trifluoroacetylacetone
FABE	Fatty acid butyl esters
FAME	Fatty acid methyl esters
FCD	Food composition database
FCT	Food composition tables
FFA	Free fatty acid
FID	Flame ionization detection
FLD	Fluorescence detector
FMOC	9-fluorenylmethyl chloroformate
FOS	Fructo-oligosaccharides
FSV	Fat-soluble vitamin
GABA	γ-aminobutyric acid
GC	Gas chromatography
GCE	Glassy carbon electrode
GCPE	Glassy carbon paste electrode
GLC	Gas-liquid chromatography
Gln	Glutamine
GLP	Good laboratory practice
Glu	Glutamic acid
Gly	Glycine
HCl	Hydrochloric acid
His	Histidine
HPLC	High performance liquid chromatography
HRMS	High resolution mass spectrometry
IAA	Indispensable amino acids
ICP-MS	Inductively coupled plasma-mass spectrometer
ICP-OES/AES	Inductively coupled plasma–optical emission spectrometry/atomic emission spectrometry
IEC	Ion-electron chromatography
Ile	Isoleucine
IR	Infrared radiation
KOH	Potassium hydroxide
L-AA	L-ascorbic acid
LC	Liquid chromatography
Leu	Leucine
LIF	Laser-induced fluorescence
LLE	Liquid-liquid extraction
LOQ	Limit of quantification
LOD	Limit of detection
Lys	Lysine
MAE	Microwave-assisted extraction
m.c.	Moisture content
MCE	Microchip electrophoresis
MD-μ-SPE	Dispersive micro-solid phase extraction
MEKC	Micellar electro kinetic chromatography
Met	Methionine
MIC	Microwave induced-combustion
MPA	Metaphosphoric acid
MRM	Multiple reaction monitoring
MS	Mass spectrometry
MS/MS	Tandem mass spectrometry
MSPE	Magnetic solid phase extraction
MTBE	Methyl tertiary-butyl ether
MTBSTFA	*N*-methyl-*N*-(tert-butyldimethylsilyl trifluoroacetamide
MUFA	Monounsaturated fatty acids
MW	Microwave heating
MW-AD	Microwave assisted-digestion
NAA	Neutron activation analysis
Nano-LC	Nano-liquid chromatography
NARP	Non-aqueous reversed-phase
NBD-Cl	4-chloro-7-nitro-2,1,3-benzoxadiazole
NEAA	Non-essential amino acid
NIR	Near infrared reflectance
NMR	Nuclear magnetic resonance
NP	Normal phase
OPA	O-phthaldialdehyde
Orn	Ornithine
PAA-T	Tetracycline-grafted polyacrylamide polymer
PCA	Perchloric acid
PDA	Photo-diode array
Phe	Phenylalanine
PITC	Phenylisothiocyanate
PLE	Pressurized liquid extraction
Pro	Proline
PUFA	Polyunsaturated fatty acids
QC	Quality control
QTOF-MS	Quadrupole time-of-flight mass spectrometry
RID	Refractive index detector
RITDF	Rapid integrated total dietary fiber
RP	Reversed-phase
SEC	Size-exclusion chromatography
Ser	Serine
SFA	Saturated fatty acids
SFC	Supercritical fluid chromatography
SNR	Signal-to-noise ratio
SPE	Solid phase extraction
SWASV	Square wave anodic stripping voltammetry
SWAdSV	Square wave adsorptive stripping voltammetry
TCA	Trichloroacetic acid
TDF	Total dietary fiber
THGA	Transverse heated graphite tube
Thr	Threonine
TMS-DM	Trimethylsilyl-diazomethane
Trp	Tryptophan
Tyr	Tyrosine
U	Expanded uncertainty
UAE	Ultrasound assisted extraction
UHPLC	Ultra-high-performance liquid chromatography
UPC^2^	Ultra-performance convergence chromatography
UV/Vis	Ultraviolet/visible
Val	Valine
VUV	Vacuum ultraviolet
WSV	Water soluble vitamin
